# On the pH Dependence of the Potential of Maximum Entropy of Ir(111) Electrodes

**DOI:** 10.1038/s41598-017-01295-1

**Published:** 2017-04-28

**Authors:** Alberto Ganassin, Paula Sebastián, Víctor Climent, Wolfgang Schuhmann, Aliaksandr S. Bandarenka, Juan Feliu

**Affiliations:** 1 Analytical Chemistry - Center for Electrochemical Sciences (CES), Ruhr-Universität Bochum, Universitätsstr, 150, 44780 Bochum, Germany; 2 Instituto de Electroquímica, Universidad de Alicante, Apartado 99, E-03080 Alicante, España Spain; 3 Energy Conversion and Storage – ECS, Physik-Department, Technische Universität München, James-Franck-Straße 1, 85748 Garching, Germany

## Abstract

Studies over the entropy of components forming the electrode/electrolyte interface can give fundamental insights into the properties of electrified interphases. In particular, the potential where the entropy of formation of the double layer is maximal (potential of maximum entropy, PME) is an important parameter for the characterization of electrochemical systems. Indeed, this parameter determines the majority of electrode processes. In this work, we determine PMEs for Ir(111) electrodes. The latter currently play an important role to understand electrocatalysis for energy provision; and at the same time, iridium is one of the most stable metals against corrosion. For the experiments, we used a combination of the laser induced potential transient to determine the PME, and CO charge-displacement to determine the potentials of zero total charge, (E_PZTC_). Both PME and E_PZTC_ were assessed for perchlorate solutions in the pH range from 1 to 4. Surprisingly, we found that those are located in the potential region where the adsorption of hydrogen and hydroxyl species takes place, respectively. The PMEs demonstrated a shift by ~30 mV per a pH unit (in the RHE scale). Connections between the PME and electrocatalytic properties of the electrode surface are discussed.

## Introduction

With the inevitable advent of the “hydrogen economy”, better understanding of electrocatalytic reactions taking place in various energy conversion and storage devices is crucial. Recent noticeable breakthroughs in fundamental understanding of electrocatalysis have been particularly accomplished *via* studies of single crystal surfaces and their activities^1–6^. Indeed, for many reactions which occur in e.g. fuel cells, such as hydrogen oxidation reaction (HOR), formic acid oxidation, ethanol oxidation reaction (EOR) or oxygen reduction reaction (ORR), not only the chemical nature of atoms at the surface but also their coordination influences the surface reactivity through different adsorption properties with respect to reaction intermediates^7–10^. Moreover, the electrode surface does not completely determine all the properties of the electrochemical interface. Electrolyte composition contributes to the performance of electrocatalytic centers, frequently in a non-trivial manner^11^. Therefore, without multi-parametric experimental probing, it might be extremely difficult to have a comprehensive picture of the processes taking place in the aforementioned energy conversion devices.

Ir is the most resistant to corrosion among the platinum group metals^12, 13^. It is also similar in many chemical and physical properties to Pt. For instance, metallic Ir has been found to be highly active for HOR in alkaline electrolytes^14^ and suitable catalyst for formic acid oxidation^15^. Inversely, its higher (compared to Pt) oxophilicity has been generally proven to be a determining factor on the suppression of EOR and ORR in alkaline solution, where OH_ad_ acts as an intermediate^16^. Potentially, a combination of the natural properties (e.g. one of the best stabilities) and a proper coordination/alloying approach would enable a rational design of stable and active electrocatalytic materials. However, basic electrochemical properties of Ir electrodes are much less investigated and consequently less understood. Several investigations have been performed on e.g. the basal planes of Ir crystals to understand its electrochemical characteristics^17–24^. Nevertheless, the lack of knowledge on Ir electrochemistry, especially in acidic environments, hinders further optimizations of electrocatalysts based on this metal and consequently its wider application in material science.

One of the feasible experimental methods to study the electrode/electrolyte interactions selectively at the interface is the laser-induced potential transient (LIPT) method^25–28^. This technique is based on a sudden increase of the temperature at the electrode/electrolyte interface by applying a laser pulse in the order of nanoseconds and permits investigations of the orientation of interfacial water *in* situ^28^. When the electrode surface is “negatively charged”, the water molecules and electrolyte components are effectively oriented at the surface with the positive dipole end toward the electrode. Increasing the temperature diminishes the orientation of dipoles, resulting in a negative potential transient. Conversely, positive transients are recorded when the surface is positively charged. The potential at which the transient changes the sign corresponds to the potential of maximum entropy (PME) of the double layer formation. This can be directly traced by the transient measurements.

As water dipoles interact electrostatically with the electric field at the interface, the PME is related to the potential of zero charge (E_PZC_), whose knowledge is a key requirement for a detailed understanding of the nature of double layer^29–33^.

The definition of E_PZC_ requires distinction of the concepts of total and free charge. These concepts are clearly defined in the case of adsorption processes, e.g. in platinum group metals, where hydrogen and hydroxyl adsorption occurs at the electrode surface. The free charge is related to the true excess of charges on each side of the interphase, whereas the total charge also includes the charge involved in the adsorption processes. Two different kinds of E_PZC_, the potential of zero total charge (E_PZTC_) and the potential of zero free charge (E_PZFC_) are associated with both kinds of charges. The E_PZFC_ is the one associated with the PME while the E_PZTC_ can be determined by the so-called “displacement method”, which quantifies the surface charge *via* adsorption of CO at the electrode surface^34, 35^.

However, the correlation between E_PZFC_ and PME is often complicated due to the existence of a chemical interaction between H_2_O molecules and the electrode surface. Due to this interaction, the water dipoles have a tendency of orienting with the oxygen toward the surface even in absence of an electric field^36–39^. Hence, PME and E_PZFC_ do not coincide exactly, although they are very close.

In this work, a combined investigation of Ir(111) single crystal electrodes was performed using LIPT, cyclic voltammetry and CO displacement techniques. Implications for energy related electrocatalytic reactions are presented.

## Results and Discussion

**Figure 1:**
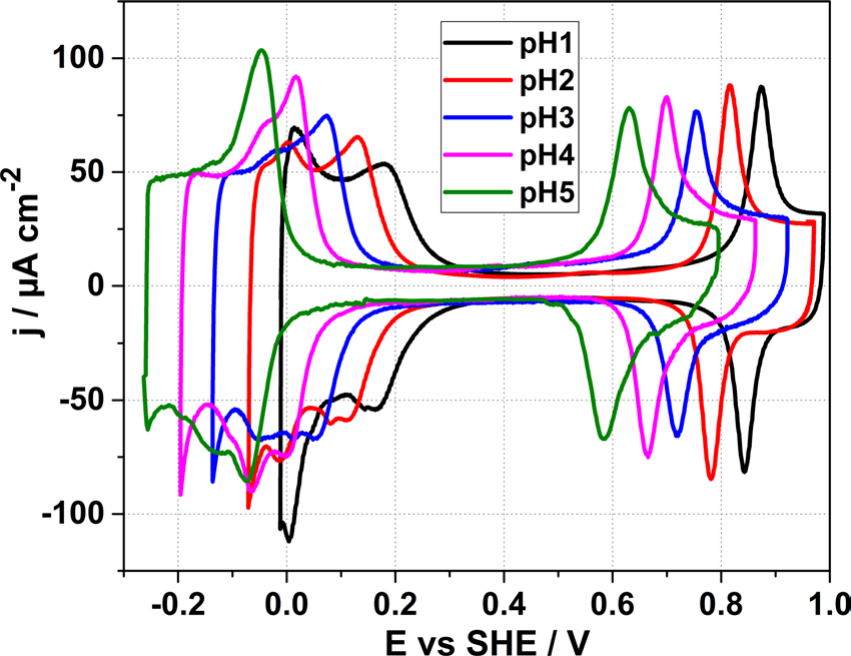
Voltammogramms of Ir(111) electrodes recorded at 50 mV/s in Ar-saturated solutions of: 0.1 M HClO_4_ (pH = 1), 0.01 M HClO_4_ + 0.099 M KClO_4_ (pH = 2), 1 mM HClO_4_ + 0.1 M KClO_4_ (pH = 3), 0.08 M KClO_4_ + 0.02 M NaF/HClO_4_ buffer electrolyte (pH = 4), and 0.1 M NaF + 1 mM HClO_4_ electrolyte (pH = 5).

In order to characterize the electrochemical behavior of the Ir(111) electrodes in aqueous electrolytes, voltammetric profiles have been recorded. In Fig. 1 the voltammetric profiles of Ir(111) in aqueous solutions with pHs from 1 to 5 are depicted.

The voltammogram recorded in 0.1 M HClO_4_, Fig. 1, is very close to those previously reported in the literature^15, 22–24^, proving the quality of the surface. At first glance, it looks fairly similar to that of Pt(111) in the same solution^22^; with a hydrogen adsorption region at low potentials followed by a double-layer region, and, at more positive potentials, a peak related to the hydroxide-ion adsorption.

The voltammogram is not symmetrical with respect to the potential axis. The characteristic features show irreversibility in the reverse scan, evincing that the hydrogen and hydroxide adsorption processes are slower than on Pt(111). The voltammetric profiles shift by −60 mV/pH. The solutions are kept at the same ionic strength by adding appropriate quantities of KClO_4_ for pH = 2 and pH = 3. Non-specifically adsorbing NaF solutions have been used as buffer electrolytes at pH = 4 and pH = 5 to prevent local pH changes near the interface. The charge associated with the peak at ~0.93 V *vs* RHE changes between 70 and 80 µC cm^−2^. The region between the lower potential of the voltammetric scan and the double-layer region presents distinctive features, which vary with the pH value. Its charge slightly decreases from ~280 µC cm^−2^ at pH = 5 to ~278 µC cm^−2^ at pH = 4, to ~260 µC cm^−2^ for pH = 2 and pH = 3 and finally to ~250 µC cm^−2^ at pH = 1. Only the latter shows a good agreement with the charge associated with a hydrogen monolayer on Ir(111), 252 µC cm^−2^ ^18^.

**Figure 2:**
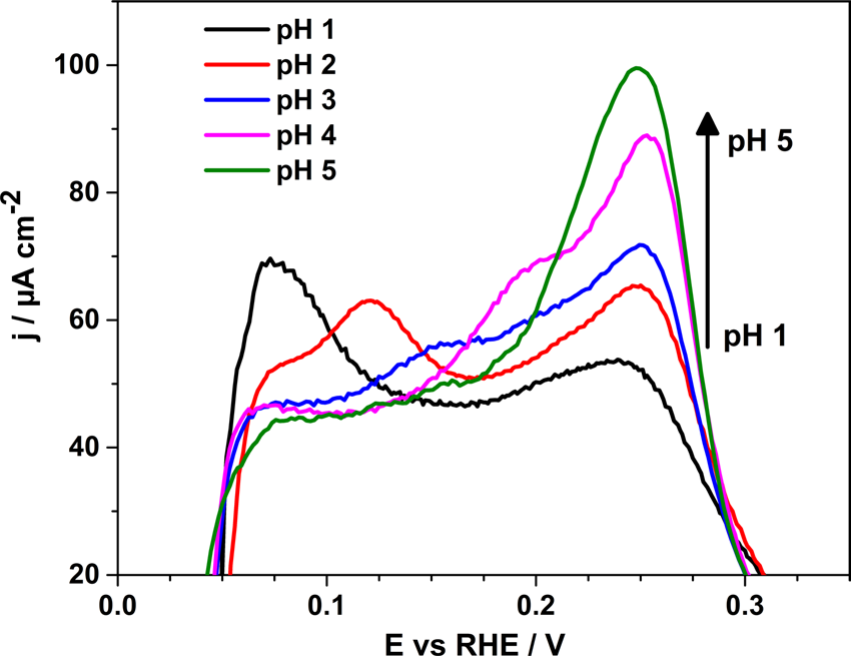
Anodic parts of the voltammograms presented in Fig. 1 (H-upd region) for the electrolytes with the pH values from 1 to 5. The arrow shows the increase of the current of the peak at ~0.25 V *vs* RHE with the pH increase.

In Fig. 2, the potential window between 0 and 0.35 V vs RHE is enlarged to highlight some distinctive features of the voltammogramms taken in HClO_4_ solutions and shown in Fig. 1.

Two peaks can be distinguished clearly in the voltammogram corresponding to pH = 1: at ~0.07 V and at ~0.25 V vs RHE. The first peak is associated with the hydrogen oxidative desorption. It shifts by ~46 mV from pH** = **1 to pH** = **2, by ~42 mV from pH** = **2 to pH** = **3 and by ~38 mV from pH** = **3 to pH** = **4. Finally, due to a further shift in the potential, the peak associated with hydrogen desorption is not visible at pH = 5 because it merges with the second peak at ~0.25 V *vs* RHE. The potential of the peak at ~0.25 V *vs* RHE doesn’t change significantly in the RHE scale if the pH of the solution increases. Nevertheless, the charge related to the peak increases monotonically. This could be an indication that hydroxyl adsorption takes place at these potentials.

**Figure 3:**
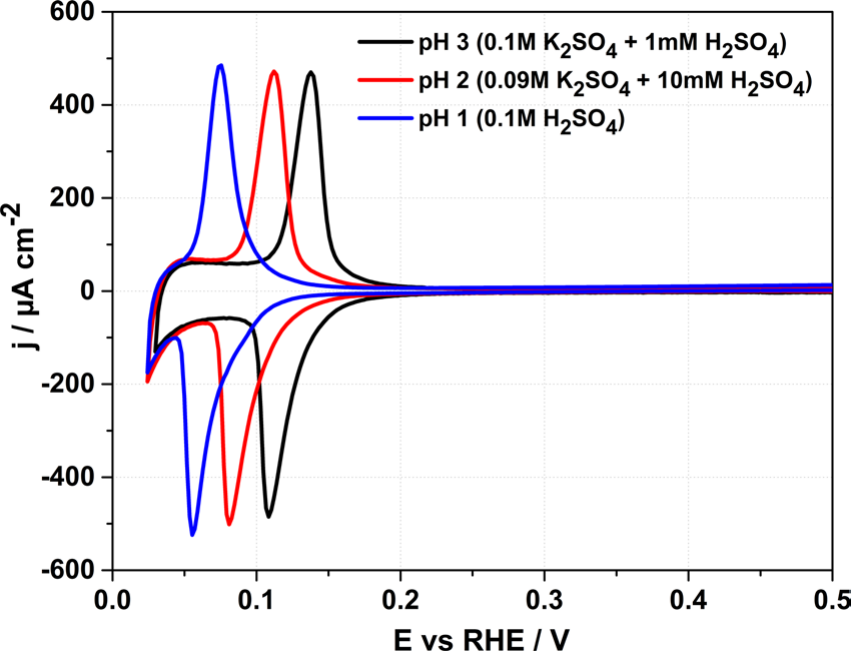
Typical voltammetric profiles for the Ir(111) electrodes in 0.1 M H_2_SO_4_ (pH = 1), 0.09 M K_2_SO_4_ + 10 mM H_2_SO_4_ (pH = 2), and 0.1 M K_2_SO_4_ + 1 mM H_2_SO_4_ (pH = 3). Scan rate: 50 mV/s (Ar-saturated electrolytes).

The voltammetric profiles for the Ir(111) electrodes in sulfate acidic solution are shown in Fig. 3.

The current interpretation of the voltammograms shown in Fig. 3 is that the characteristic peak that can be seen between 0.05 and 0.15 V *vs* RHE at pH = 1 corresponds to coupled hydrogen desorption and sulfate adsorption. During the anodic sweep, sulfate replaces the hydrogen adatoms and covers the Ir(111) surface until irreversible oxide formation occurs (~1.35 V vs RHE)^18^. Ito *et al*.^40^ and Itaya *et al*.^18^ confirmed the presence of adsorbed structures in 0.1 M H_2_SO_4_ electrolyte at potential higher than the sulfate peak using scanning tunnelling microscopy (STM) and infrared adsorption spectroscopy (IRAS). Results obtained via spectroscopic methods reveal vibrational modes which are characteristic for the specific adsorption of sulfate ions^40^. STM measurements have shown that the adsorbed sulfates form an ordered √3 × √7 structure on the Ir(111) surface^18^.

Integration of the charge corresponding to the peaks shown in Fig. 3 for the 0.1 M H_2_SO_4_ solution gives ~272 µC cm^−2^, slightly higher than the theoretical value of 252 μC cm^−2^ corresponding to the hydrogen adlayer with a 1:1 ratio of *H to Ir atoms involving a one-electron-transfer reaction at an ideal Ir(111) − (1 × 1) surface.

At pHs = 2 and 3 the ionic strength was kept constant by adding K_2_SO_4_. The charge correlated to the sulfate adsorption peak corresponds to ~267 µC cm^−2^ at pH = 2 and to ~270 µC cm^−2^ at pH = 3. Taking into account various sources of uncertainties, it can be affirmed that the charge remains constant.

In order to clarify the nature of the adsorption processes at the H-upd region, LIPT measurements were performed. LIPT allows the estimation of the PME also for non–ideal polarizable surfaces like Pt or Ir, thus providing valuable information regarding the Metal(h k l) | solution interface.

**Figure 4:**
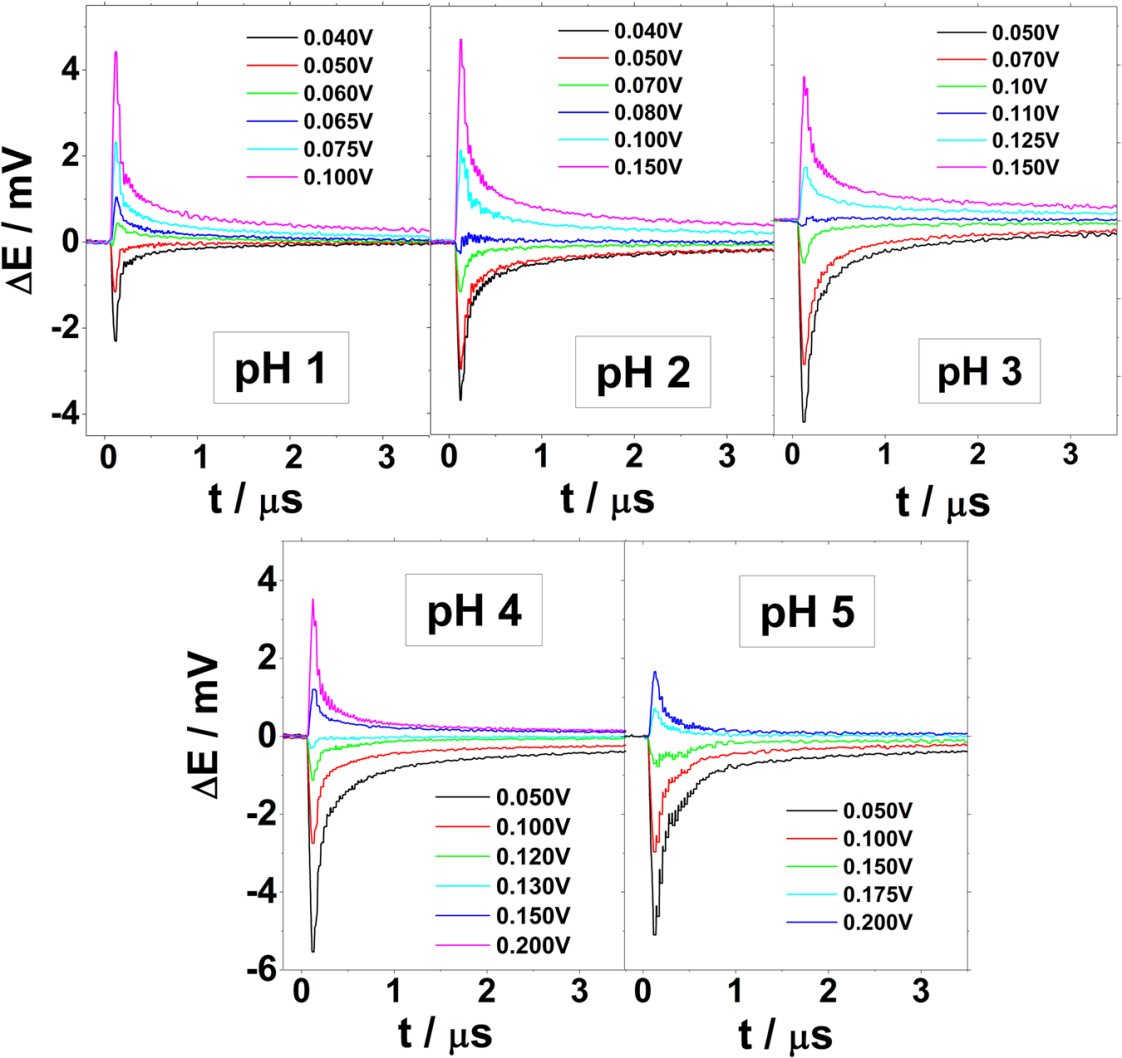
Laser-induced potential transients for Ir(111) at different pHs. Laser beam energy: 1 mJ/pulse. All the potentials are given *versus* the RHE scale. Composition of the electrolytes is the same as in Fig. 1.

Figure 4 shows the potential transients recorded for Ir(111) in the perchlorate/fluoride electrolytes with pHs from 1 to 5. When the recorded transients are negative, the thermal coefficient of the double layer potential drop is negative (dE^M^/dT < 0), and the water molecules are oriented with the pair of hydrogens pointing towards the surface. By increasing the applied potentials sufficiently, the magnitude of the laser induced potential transient decreases until it reverses sign. When the thermal coefficient is positive, the water molecules are preferably oriented with the oxygen towards the surface. Remarkably, within the overall potential window, the transient profiles show a monotonous decay after the sharp increase caused by the laser irradiation. Such monotonic profile indicates that the recorded transient response is just due to the double layer reorganization. Non monotonous responses have been observed for Pt and Au when rate of adsorption process is enough to compete with the double layer reorganization^27, 41^. Even at the more acidic pH, either at pH = 1 or pH = 2, the laser induced potential transients recorded at low potentials (H-upd region) still show monotonic profiles. That allows to determine the PME more precisely since no other contributions overlaps with the purely capacitive response. On the other hand, at both pH = 1 and 2, the transients recorded for Pt(111) have bipolar profile, thus evidencing more than one contribution to the temperature perturbation (see Supplementary Fig. S1). The most positive and slower currents (higher relaxation time) were assigned to a shift of the hydrogen adsorption equilibrium, whereas the negative and faster current contribution were related to the relaxation associated with the double layer itself. At those pH values, the hydrogen adsorption on Pt(111) is fast enough to cause a positive shift of the electrode potential in the time scale employed^41^. The rate of this reaction decreases when pH is increased. For Pt(111), above pH = 3, the proton adsorption contribution is completely decoupled from the overall laser response. Therefore, the monotonic laser response recorded on Ir(111) evidences that the hydrogen adsorption process is slower on Ir(111) than on Pt(111). This result is in agreement with the recorded voltammetric profiles and also with the impedance measurements performed by Kolb *et al*.^24^.

**Figure 5:**
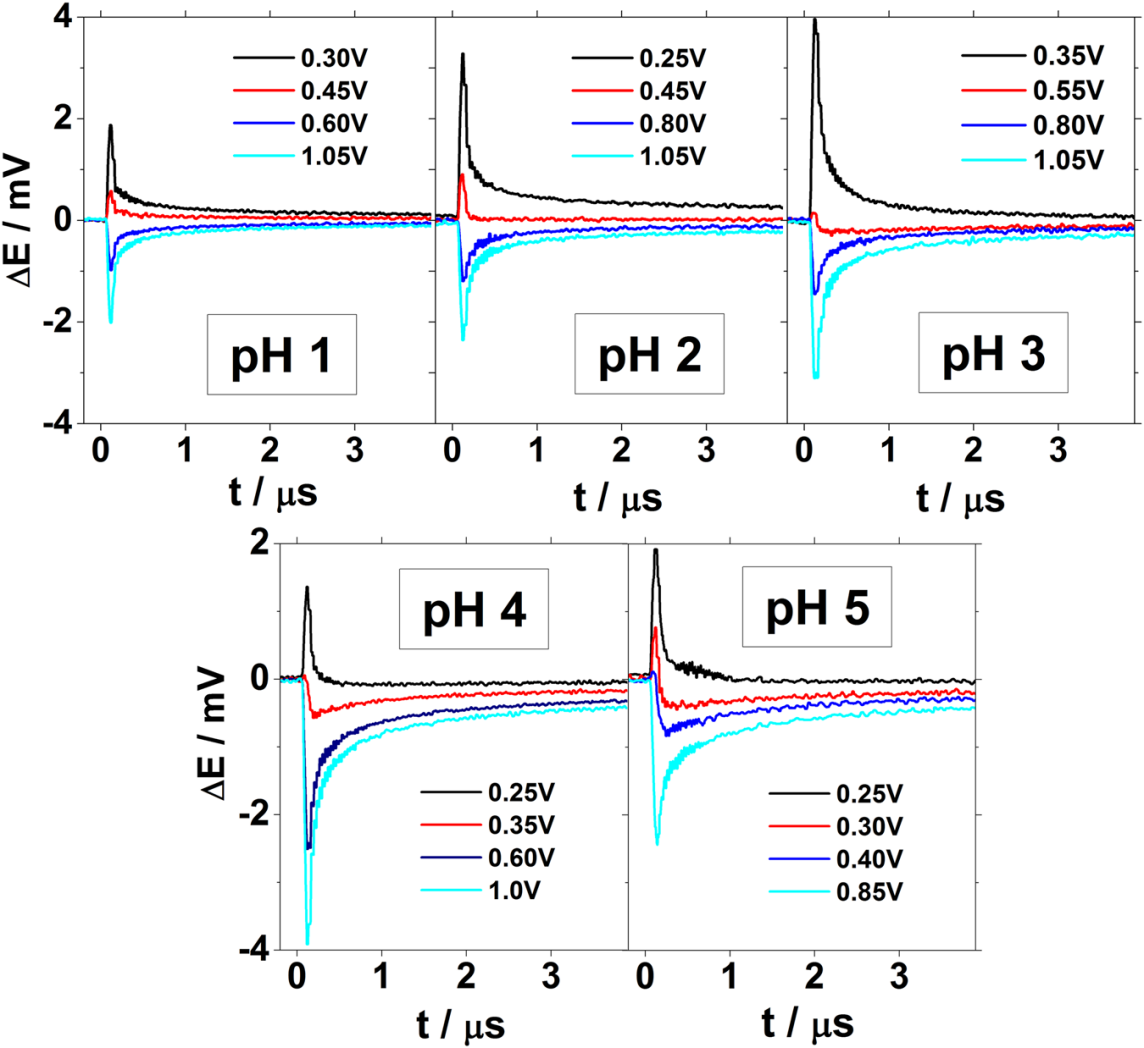
Laser induced potential transients for Ir(111) recorded at the potentials more positive than PME at different pHs. Laser beam energy: 1 mJ. All potentials are given versus the RHE scale. Composition of the electrolytes is the same as in Fig. 1.

Figure 5 shows the laser induced potential transients recorded for the same perchorate/fluoride solutions but at more positive potentials, above the PME. At potentials between 0.4 V and 0.6 V *vs* RHE the recorded laser induced potential transients reverse sign from positive to negative. This second change of the sign of the transients was previously observed in Pt(111)^41^, although at higher potential values, and it is an indication that the surface is covered by anions. The presence of anions changes the surface polarization causing a re-orientation of the water molecules. Since neither fluorides nor perchlorates adsorb specifically at the Ir(111) surface, hydroxide-ions are the species which are likely adsorbed, causing the reversal of the sign of the transients. It is remarkable that the change in the transient sign takes place at potentials much lower than 0.93 V *vs* RHE, the potential value where the broad peak, previously assigned to hydroxide adsorption^24^ is recorded in the cyclic voltammogramms in Fig. 1. Therefore, this result suggests that hydroxide adsorption could take place at lower potentials than those expected from the voltammetric profiles.

The PME is estimated by calculating the temperature coefficient at each applied potential. From the electrocapillary equation^42^, for a sufficiently fast temperature change, it can be shown that^36^:

1 $${({{\rm{dE}}}^{{\rm{M}}}/{\rm{dT}})}_{{\rm{q}}}=-{({\rm{d}}{\rm{\Delta }}S/{\rm{dq}})}_{{\rm{T}}}$$

where q is the free charge density on the metal and ΔS is the entropy of formation of the double layer. According to this equation, at PME (dΔS/dq)_T_ = 0, the temperature coefficient of the double layer is zero. To calculate the thermal coefficient, the LIPT linearization of the transient data was performed by plotting them as a function of 1/t^0.5^ (see supplementary information for the calculation details).

**Figure 6:**
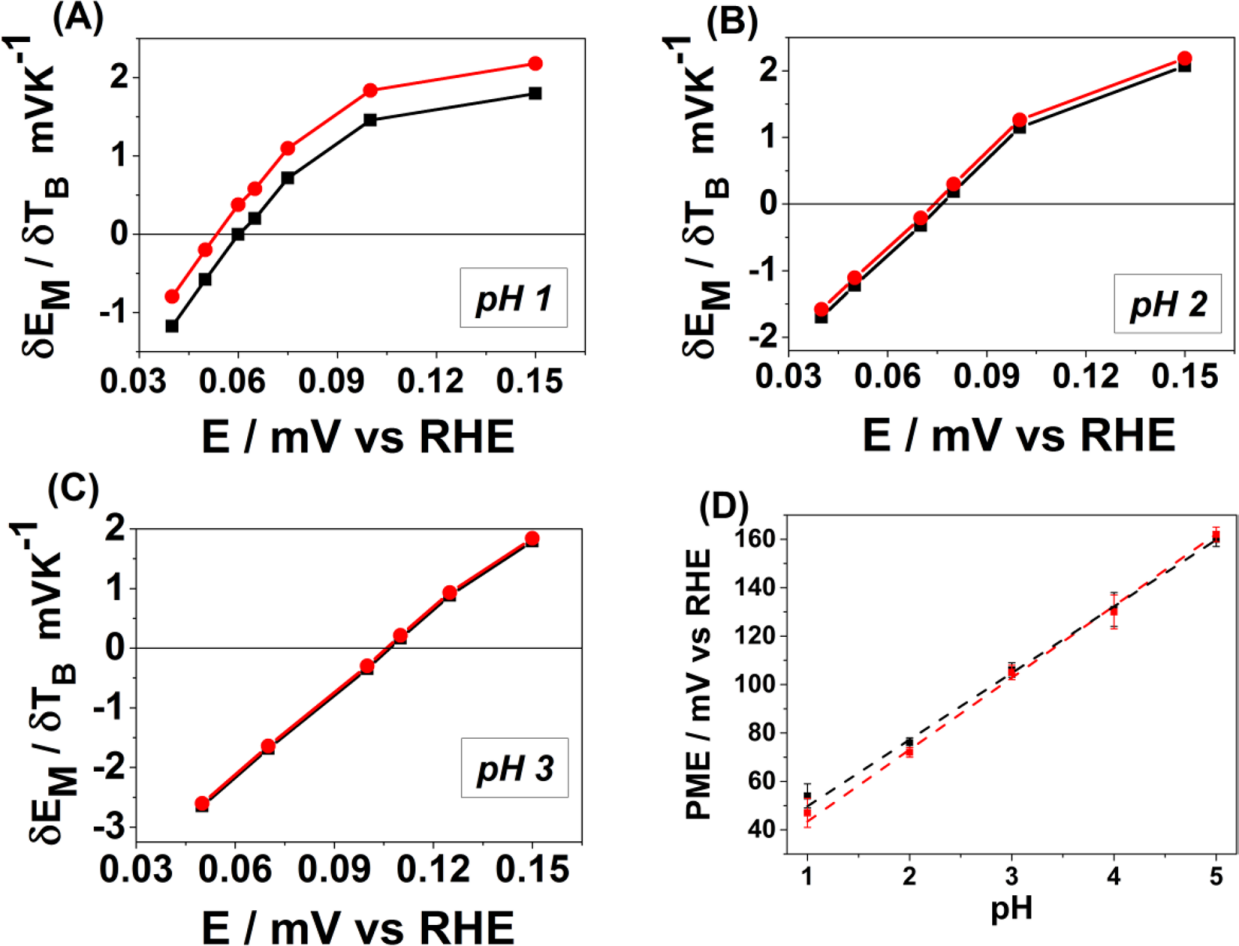
Temperature coefficient of the double layer potential with (red circles) and without (black squares) the thermodiffusion correction for Ir(111) at pH = 1 (**A**), pH = 2 (**B**) and pH = 3 (**C**) respectively. (**D**) Plot showing the PME values calculated at different pHs with (red symbols) and without (black) the thermodiffusion correction.

Figure 6(A,B and C) show the (dE^M^/dT)_q_ *vs* E plots for the Ir(111) electrodes characterized in the electrolytes with pHs from 1 to 3 with and without the thermodiffusion potential correction. The latter arises in a solution as a consequence of the temperature difference between working and reference regions. The PME is located at the potential where the graph crosses the x-axis. The thermodiffusion potential can be estimated by knowing the Eastman entropies of transport for different ions in the electrolyte^43^. The solution at pH = 1 shows the largest thermodiffusion potential contribution due to the higher mobility of protons compared to the other ions in solution. For pH > 3 the thermodiffusion potential is essentially negligible due to the lower proton concentration. Table 1 shows the thermodiffusion potentials for different electrolytes used in this work.

**Table 1:** Calculated thermodiffusion potentials for the electrolytes employed in this work using the transport numbers calculated from the ionic mobilities at infinite dilution^71^.

Solution	[ΔE_Thermodiffusion_/ΔT]/mV K^−1^
0.1 M HClO_4_ (pH = 1)	−0.381
10 mM HClO_4_ + 0.09 M KClO_4_ (pH = 2)	−0.115
1 mM HClO_4_ + 0.099 M KClO_4_ (pH = 3)	−0.048
0.08 M KClO_4_ + 0.02 M NaF/HF (pH = 4)	−0.030
0.1 M NaF + 1 mM HClO_4_ (pH = 5)	0.0264

Figure 6(D) shows the dependencies of the calculated PME as a function of pH in the range from 1 to 5. The PME clearly shifts by ~30 mV per pH unit. This result is in contrast to the calculated PME for Pt(111), which remains constant in the SHE scale^41^. Moreover, the estimated PME at pH = 0 is extremely low, approximately 8 mV *vs* SHE, whereas the extrapolated PME value of Pt(111) is around 300 mV *vs* SHE. This result indicates that despite the chemical and physical similarity between Pt(111) and Ir(111) electrodes, their PME values, and hence interfacial properties, show huge dissimilarities when in contact with aqueous solutions.

It should be highlighted that the PME is located within the H-upd region in the studied pH range suggesting that even within the double layer region the Ir(111) electrodes remain positively charged. It is worth pointing out that the voltammetric profile in the H-upd region is the most sensitive to the pH change if compared to the rest of the voltammogram (see Fig. 2). At lower pH values, the two peaks in the H-upd region described previously shifts until they overlap to result in one big peak centered at 250 mV at pH = 5. Thus, all these experimental observations suggest that the “H-upd region” does not only involve hydrogen adsorption, but the specific adsorption of hydroxide-ions as well.

**Figure 7:**
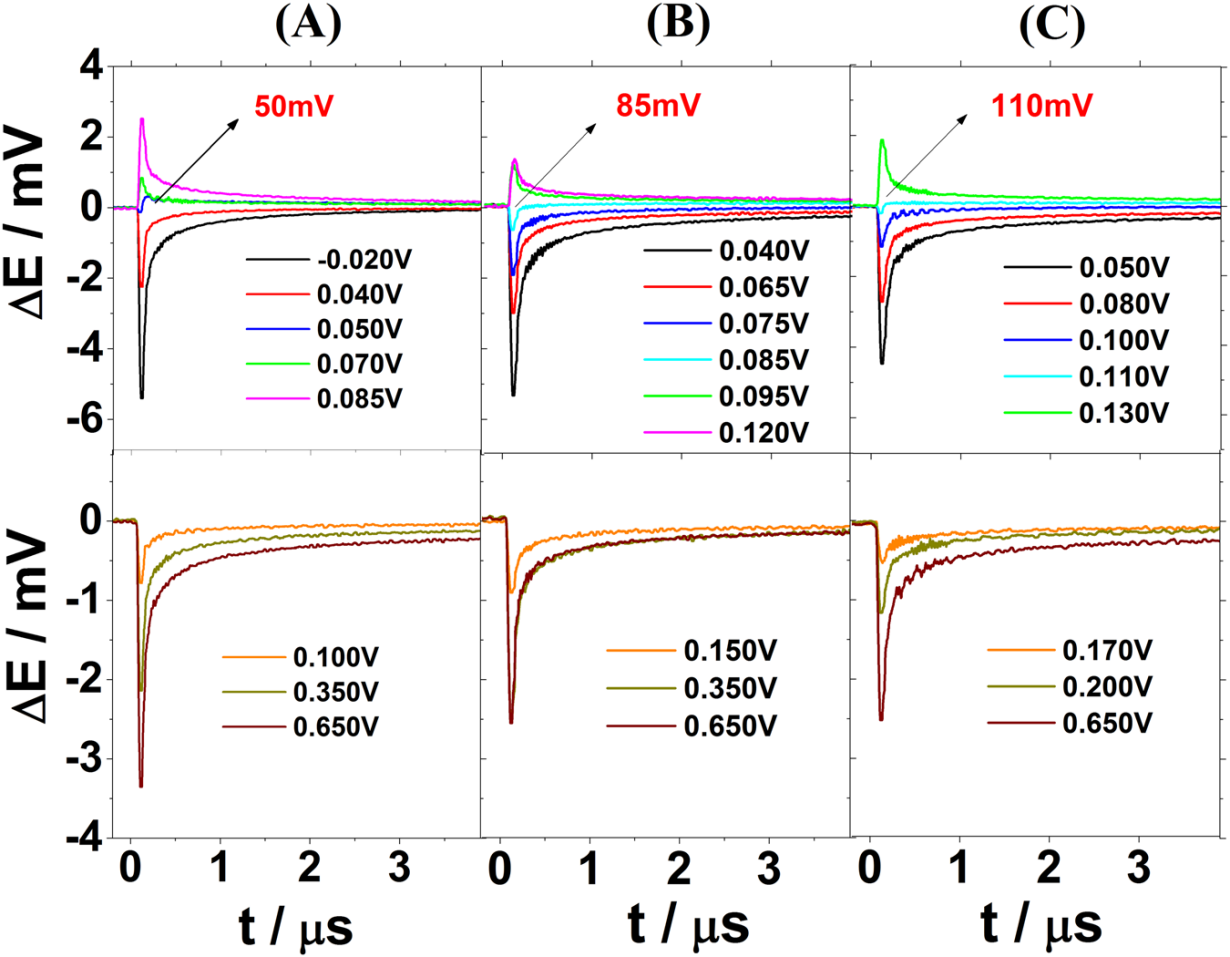
Laser induced potential transients for Ir(111) electrodes in contact with (**A**) 0.1 M H_2_SO_4_, (**B**) 0.01 M H_2_SO_4_ + 0.09 M K_2_SO_4_ and (**C**) 1 mM H_2_SO_4_ + 0.1 M K_2_SO_4_. PME values are highlighted in bold red in the upper part of the figures. Laser beam energy: 1 mJ. All given potentials are referred to the RHE scale.

The interface between Ir(111) and sulfuric acid electrolytes was also investigated by means of LIPT in order to elucidate the influence of sulfate specific adsorption on the restructuration of the interfacial H_2_O-network. Figure 7 shows the laser induced potential transients for Ir(111) electrodes in contact with sulfate containing electrolytes at pH = 1, pH = 2, and pH = 3. Our measurements show that in these systems the PME is located at the onset of the sulfate adsorption and shifts by around 30 mV in the RHE scale per pH unit, similar to the voltammetric peaks (see Figs 3 and 7 respectively). Thus, the adsorption of sulfate-anions takes places when the surface is positively charged, if we accept the correlation between PME and E_PZFC_. The potential transients, however, become negative again at ~100 mV at pH = 1, ~140 mV at pH = 2 and ~170 mV at pH = 3 in the RHE scale, therefore after the large peak related to sulfate adsorption (see Figs 3 and 7). The change of the sign of the transients takes place at much lower potentials in the sulfate/sulfuric electrolytes compared to perchlorate/fluoride ones, indicating that the sulfate-surface interactions are stronger than interactions between the hydroxide-ions and Ir(111). Therefore, the adsorption of sulfate-anions causes a strong change on the surface polarization reorienting the water molecules. An analogous effect of sulfate adsorption on the interfacial water restructurisation was previously reported for Au(111) and Pt(111) electrodes^27, 28^.

In order to further elucidate the nature of the adsorption-desorption processes at the Ir(111)/electrolyte interface, CO-displacement experiments were performed. CO-displacement is known to be an effective strategy of determining the E_PZTC_ as it measure the change in surface charge at a given electrode potential during the effective “quenching” of the double layer by CO adsorption. The E_PZTC_ is then defined as the potential at which the total surface charge of the metal is equal to 0. CO-displacement experiments performed by Kolb *et al*.^24^ in 0.1 M H_2_SO_4_ showed that the E_PZTC_ is located at the center of the voltammetric peak at ~0.1 V *vs* RHE. On the other hand, as discussed above, the voltammetric profiles in HClO_4_ electrolytes are not yet fully understood, and CO-displacement experiments can help in further elucidation of the nature of the adsorption properties of those systems.

For each CO-displacement the electrode potential was fixed and CO gas was flowed through the solution. The CO molecules were adsorbed at the surface and the current transients related to the displaced species were recorded. After integration of those transients, the surface charge at the chosen potential can therefore be determined.

The charge measured in such an amperometric experiment at constant potential during CO adsorption corresponds to the difference between the charge density on the CO-covered and the CO-free surfaces:

2 $${{\rm{q}}}_{{\rm{d}}}={{\rm{q}}}_{{\rm{m}}/{\rm{CO}}}\,\mbox{--}\,{{\rm{q}}}_{{\rm{m}}}$$

where q_d_ is the charge displaced during the potentiostatic adsorption of CO, q_m/CO_ is the charge density for the CO-covered electrode, and q_m_ is the charge density for the CO-free surface. The double layer capacitance of the CO-saturated Ir(111)-surface is markedly smaller than that observed in the absence of CO as measured by cyclic voltammetry. Therefore, as a first approximation, q_m/CO_ can be considered negligible^44^.

The displaced charge during the potentiostatic adsorption can therefore be approximated as the charge density at the surface of the metal.

q_m_ is derived from the following equation:

3 $${{\rm{q}}}_{{\rm{m}}}={\rm{\sigma }}-{{\rm{F}}{\rm{\Gamma }}}_{{\rm{H}}}+{{\rm{F}}{\rm{\Gamma }}}_{{\rm{OH}}}$$

where σ is the charge localised on the metal side of the double layer (so called free charge density), Γ_H_ the thermodynamic excess of the adsorbed hydrogen and Γ_OH_ the thermodynamic excess of the adsorbed OH. The value of q_m_ is therefore strongly influenced by the amount of adsorbed species. For high coverage of adsorbed species, the extent of the contribution of hydrogen and OH adsorption prevails over the contribution of the free charge density. However, the thermodynamic excess of adsorbed species cannot be measured in aqueous solutions. As can be deduced from equation (3), H adsorption compensates positive σ, and OH adsorption negative σ. Consequently, the E_PZTC_, being the potential at which q_m_ = 0, shifts to higher potential when the amount of adsorbed hydrogen is higher than that of hydroxide. As can be observed in Fig. 2, H and OH adsorption regions cannot be easily de-convoluted.

**Figure 8:**
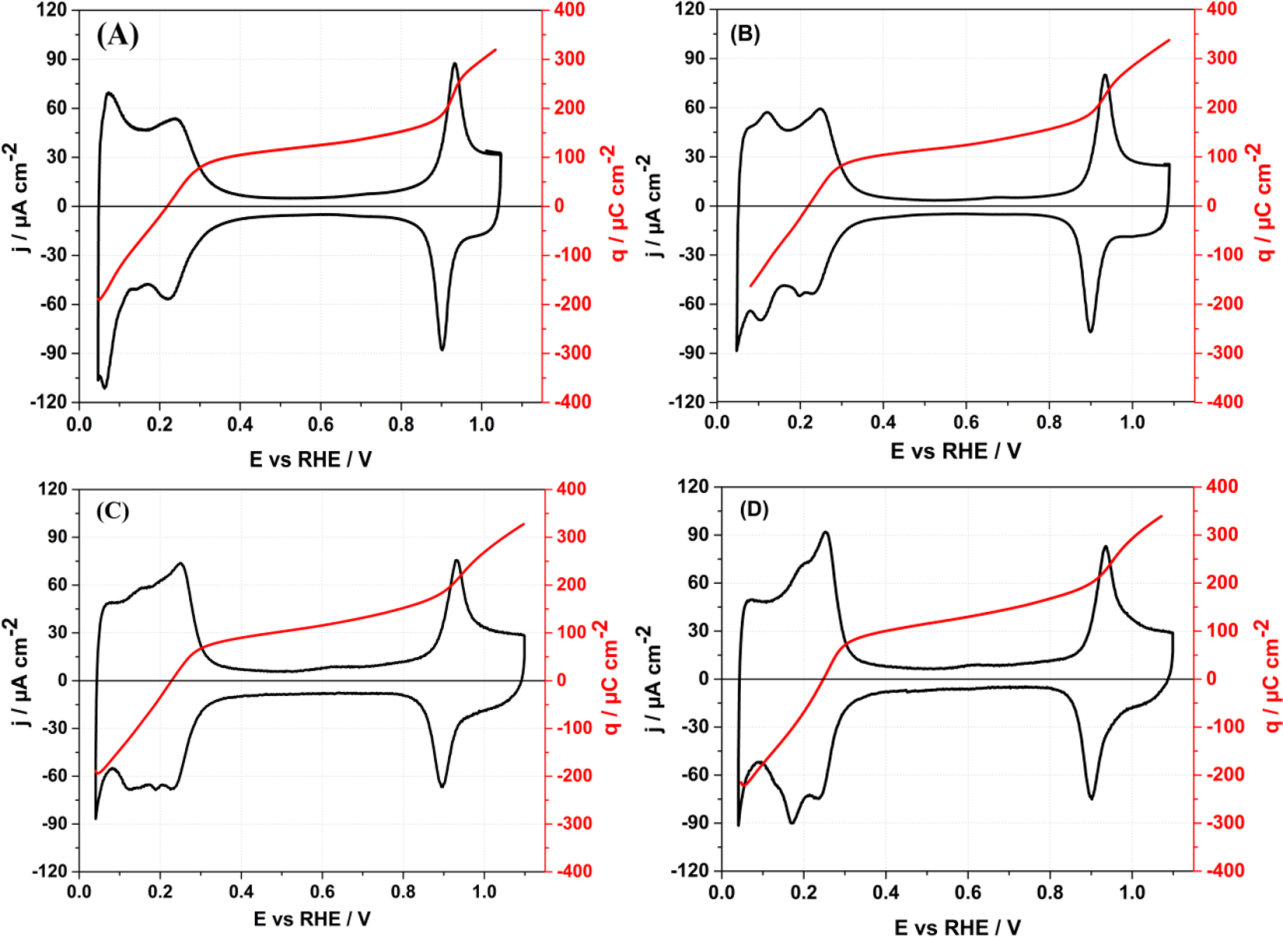
Voltammetric profiles of Ir(111) at (**A**) pH = 1, (**B**) pH = 2, (**C**) pH = 3, (**D**) pH = 4. Solutions employed are described in Fig. 1(A) and (B). Scan rate 50 mV/s.

Figure 8 depicts the cyclic voltammograms of Ir(111) obtained in the perchlorate electrolytes with pHs from 1 to 4 and the charge curves obtained from the integration of the corresponding voltammogramm with the use of the charge displaced by CO at 0.3 V using equation (4):

4 $${{\rm{q}}}_{{\rm{m}}}({\rm{E}})={\int }_{{\rm{0.3}}}^{{\rm{E}}}\frac{{\rm{j}}}{{\rm{V}}}{\rm{dE}}-{{\rm{q}}}_{{\rm{d}}}(0\mathrm{.3V})$$

Negatively charged species are reductively displaced by CO at 0.3 V *vs* RHE (see Supplementary Fig. S3), indeed indicating that OH_ad_ is adsorbed at the anodic peak centered at ~0.25 V vs RHE. The co-adsorption of hydrogen and OH takes place in the potential region where the E_PZTC_ is located.

**Figure 9:**
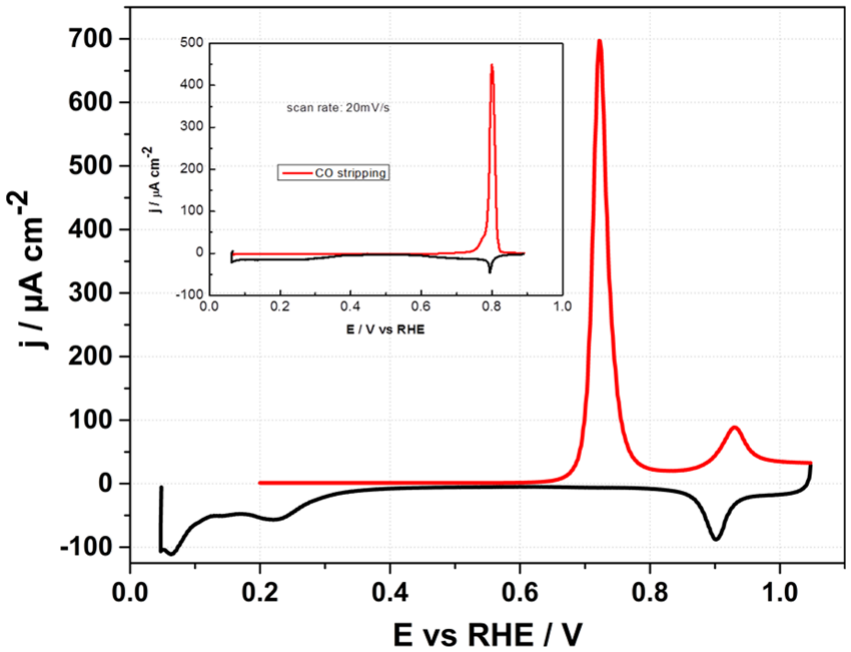
CO stripping voltammogram of Ir(111) after the CO displacement at 0.2 V vs RHE in 0.1 M HClO_4_ (pH = 1). The red line represents the CO stripping voltammogram and the black line shows the cathodic scan after the stripping. Scan rate 50 mV/s. Inset shows the corresponding voltammogram for the CO stripping from the Pt(111) electrode in 0.1 M HClO_4_.

After the CO-adsorption, the surface was recovered to the initial state *via* CO stripping (Fig. 9). It is worth to say that the CO oxidation takes place at less positive potentials on Ir(111) than on Pt(111). While the CO oxidation peak appears at ~720 mV vs RHE on Ir(111) in 0.1 M HClO_4_ solution, on Pt(111) the peak is located at ~800 mV vs RHE (inset in Fig. 9). Ir and Pt have similar atomic sizes (0.272 *vs* 0.278 nm), therefore comparing the adsorption of CO at Pt(111) and Ir(111) can provide useful insights into the electronic effects of the surfaces on the interfacial structures. The comparison of the same crystallographic plane allows decoupling between the geometric and electronic effect and the difference in activity can be attributed solely to the difference in electronic structure between the two electrocatalysts. On the other hand, it is well known that the mechanism of CO oxidation involves adsorbed OH^45, 46^. In both Ir(111) and Pt(111), the CO oxidation happens on a metal surface onto which OH is specifically adsorbed through a Langmuir Hinshelwood mechanism. Therefore, one explanation for the highest catalytic activity of Ir(111) for CO oxidation could be that the adsorption of OH on Ir(111) takes place at lower potentials compared to Pt(111) and leads therefore to a decrease of the overpotential for CO oxidation. This would be in agreement with previous results obtained by LIPT.

For the coulometric estimation of CO coverage, the charge obtained from the CO oxidation peak integration was corrected for the charge of the current–potential profile measured in the absence of chemisorbed CO according to the procedure described by Weaver *et al*.^47^. This is obtained by integrating the voltammogram between the E_PZTC_ and the upper limit of the potential scan during CO stripping. The CO oxidation charge was measured to be ~295 µC cm^−2^ in 0.1 M HClO_4_, slightly lower than the reported literature values of 300 µC cm^−2^ ^48, 49^. This value in turn corresponds to the fractional CO coverage of ϴ = 0.59 as compared to the reported values of ϴ = 0.6. The reported ϴ at pH=1 is consistent with the coverage measured upon CO stripping in 10 mM HClO_4_ + 0.09 M KClO_4_ solution (pH = 2), 295 µC cm^−2^ (see Supplementary Fig. S5). In 1 mM HClO_4_ + 0.099 M KClO_4_ (pH = 3), and in 0.08 M KClO_4_ + 0.02 M NaF/HF (pH = 4) the charge obtained upon stripping was ~312 µC cm^−2^, and ~324 µC cm^−2^, respectively (see Supplementary Figs S6 and S7).

**Figure 10:**
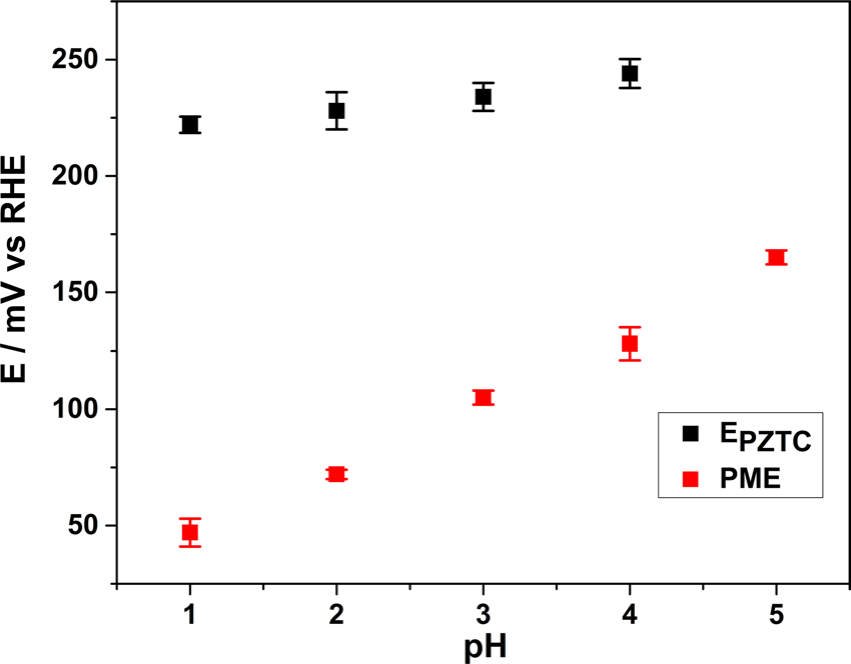
E_PZTC_ (black dots), and PME values corrected for the thermodiffusion coefficient (red dots), as a function of the proton concentration in perchloric electrolytes. The composition of the electrolytes is the same as described in Fig. 1.

Figure 10 compares E_PZTC_ and PMEs for Ir(111) electrodes at different pHs. The E_PZTC_ increases slightly against the logarithm of the proton concentration (by ~7 mV/dec). The increase in potential of the E_PZTC_ with the increase of the pH indicates a compensation of the negative free charge of the metal by the positive charge due to higher OH-coverage. Based on equation (3) and on the fact that the E_PZTC_ is higher than the PME, we can affirm that the surface excess of hydrogen is higher than that of OH at E_PZTC_ potentials. For the four pHs examined, the E_PZTC_ is situated in the H and OH coadsorption region, always before the peak in the voltammograms at ~0.25 V vs RHE corresponding to the OH-adsorption. The nature of the latter is confirmed furthermore by the fact that the potential difference between PME and E_PZTC_ increases as the pH decreases.

Water dipoles are strongly affected by the electrostatic forces with the free charge density on the surface, therefore the potential of reorientation of the water is closely related to the E_PZFC_. However, the net orientation of the water dipole is also affected by the chemical interactions between water molecules and the metal surface, especially in the case of transition metals, where the specific adsorption of H and OH takes place. In the total absence of adsorption processes, E_PZFC_, and, hence, the PME should shift by 60 mV in the RHE scale. The observed PME-shift of ~30 mV for the Ir(111) electrodes indicates that adsorption processes affect the distribution of free charges at the interphase. Unfortunately, contributions of the electrostatic forces and of the chemical metal-H_2_O interactions cannot be decoupled in aqueous solutions.

The PME has been shown to be located close to the E_PZFC_ in the case of Hg^36, 50^, Au^27, 38, 51^, and Pt(111)^52^ electrodes. Due to the existence of a charge-transfer process, the surface cannot be considered as ideally polarizable, and the conclusions drawn for Hg and Au cannot be transferred to the case of Ir(111). In the latter case, the direct measurements of the E_PZFC_ are precluded by the hydrogen and anion adsorption. For Pt(111), which specifically adsorbs H and OH as Ir(111), the extrapolation of the E_PZFC_ from the E_PZTC_ has been performed via further calculations which required the knowledge of the E_PZC_ of the CO covered surface inferred from UHV measurements. The UHV based estimation of the E_PZC_ of Ir(111) is a value not yet available for the Ir(111)-CO surface.

**Figure 11:**
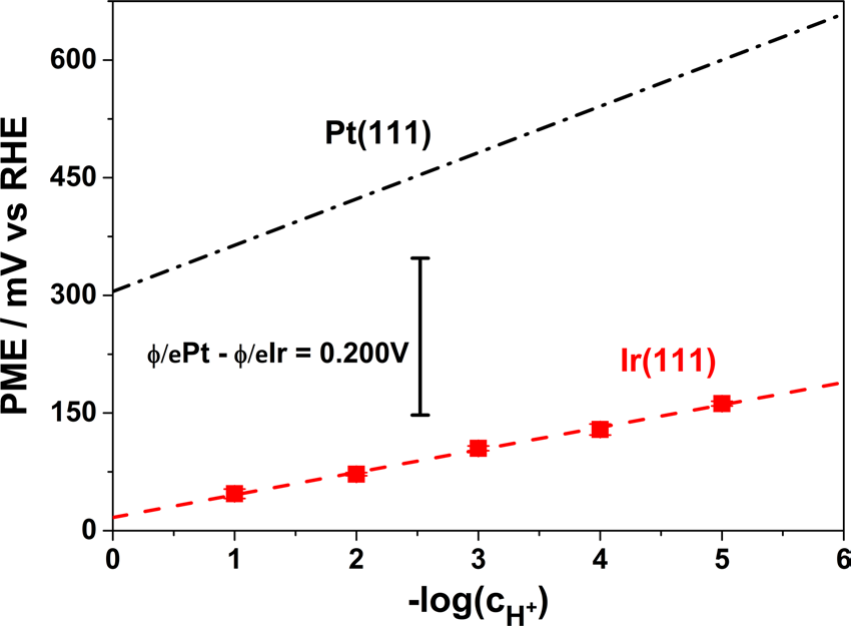
PME-trends as a function of pH elucidated in this work for Ir(111) and for Pt(111) as reported by Garcia-Araez *et al*.^41^. The difference in the work functions of Pt(111) and Ir(111) in vacuum is also shown for the sake of comparison.

Both LIPT and CO displacement measurements have shown the great influence of the electrolyte composition on the restructuration of the double layer, thus modifying the interfacial properties of Ir(111). Here, the specific nature of the anion must be considered as it plays an important role in the redistribution of the charge across the interface (either hydroxide or sulfate-anions). In particular the LIPT has provided PME values consistently lower for Ir(111) than for Pt(111). This trend qualitatively agrees with the work function values of both metals, but the potential differences between Ir(111) and Pt(111) PMEs are higher than the difference between the work functions values (*ϕ* ^*M*^). The calculated *ϕ* ^*M*^/*e* of both Pt(111) and Ir(111) in vacuum conditions differ between 70–200 mV, according to references^53, 54^. However, as can be seen in Fig. 11, the difference between their calculated PME in aqueous solution and *ϕ* ^*M*^ values is higher, and this difference increases with the pH. For comparison, Fig. 11 shows the linear variation of the PME with the pH for Ir(111) and Pt(111) (from ref. 41) in the RHE scale.

In order to understand the observed discrepancy between the PME values and metal work functions of Pt and Ir, especially when the pH is increased, it is convenient to consider different contributions to the electrode potential in the absence of ion specific adsorption^31^:

5 $${E}^{M}={\varphi }^{M}/e+\delta {\chi }^{M}+{g}^{s}(dip)+{g}_{s}^{M}(ion)-{E}_{abs}(ref)$$

where *Φ* ^*M*^ is the work function of the clean metal surface, *e* is the electron charge, *δχ* ^*M*^ is the change in the surface electron (“electron spillover”) contribution to *Φ* ^*M*^ caused by the contact with a solvent, *g* ^*s*^(*dip*) is the surface potential component due to the net solvent dipole orientation, *g*_*s*_^*M*^(*ion*) is the contribution from “free” charges, associated with excess electronic charge on the metal surface along with the ionic double-layer countercharge, and *E*_*abs*_(*ref*) is so-called “absolute” potential of the reference electrode. It is worth mentioning that, while the main contribution to the thermal coefficient comes from the dipolar layer as discussed above, other terms might also affect this parameter. One of them is the thermal coefficient for the electrode work function. Its value is usually very small, i.e. ≈0.15 mV/K for Pt(111), and for Ir(111) the *ϕ* ^*M*^ does not show any significant temperature dependence, even though the exact value of the thermal coefficient for the work function could not be estimated due to the large error bars^53^. The E_PZC_ can be obtained from equation (5) just considering that *g*_*s*_^*M*^ (*ion*) = 0:

6 $${E}_{PZC}={\varphi }^{M}/e+\delta {\chi }^{M}+{g}^{s}(dip)\,-\,{E}_{abs}(ref)$$

The *g* ^*s*^(*dip*) contribution is often regarded as constituting the major solvent influence on E_PZC_, but the interfacial electron density profile (i.e., the *δχ* ^*M*^ term in equation 6) is also modified by the solvent-induced contribution. However, although the contribution of the solvent to the potential seems to be of crucial importance, the calculation of the magnitude of *δχ* ^*M*^ as well as *g* ^*s*^(*dip*) requires understanding of solvent-induced changes on local surface potentials, which has not yet been achieved. Weaver demonstrated the importance of the *δχ* ^*M*^ and *g* ^*s*^(*dip*) terms for Pt(111) using UHV-based study of solvation-induced *Φ* ^*M*^ changes at suitably low temperatures^55^. The large (*ca* 1 eV) *Φ* ^*M*^ decrease measured on Pt(111) in UHV upon dosing water can therefore be attributed to important contributions from both the *g* ^*s*^(*dip*) and *δχ* ^*M*^ terms^39^. CO charge displacement data, combined with the known *Φ* ^*M*^ value for Pt(111), 5.9 eV, by means of equation (6), suggests the presence of a large negative interfacial solvent contribution to the electrode surface potential. In the case of Ir(111) there is no UHV-based study of solvation-induced *Φ* ^*M*^ changes. Based on the results obtained in this work, we can compare the relative contribution of *g* ^*s*^(*dip*) and *δχ* ^*M*^ to the PME measured for both metals as a function of the pH. When the pH increases, even in absence of an electric field, the magnitude of the chemical interaction between H_2_O molecules and the electrodes surfaces is described by a linear correlation having different slopes for Ir(111) and Pt(111). These observations would agree with the highest oxophilicity of Ir compared with Pt reported by Markovic *et al*.^14^, since a stronger interaction of the Ir(111) with the solvent is expected from the obtained results.

The comparison of the basic electrochemical properties of the same crystallographic plane of two metals belonging to the same period and block (i.e. 6d), and having the same *fcc* crystal structure, similar mass and atomic radius, can give fundamental insights into the mechanism of electrocatalytic reactions.

As demonstrated above, the same surface charge for the Pt(111) and Ir(111) do not correspond to the same electrode potential, owing to the different metal-water interactions. These changes in the interfacial electric field, and electrode Fermi level, modify the water structure and alter the reaction barrier for electrocatalytic reactions, as shown in this work for CO electro-oxidation. The comparison between the two surfaces can be used to gain mechanistic insights for important electrocatalytic reactions, for instance the methanol oxidation reaction (MOR). The MOR on Pt(111) proceeds via two possible paths involving either adsorbed methoxy (CH_3_O_(ad)_) or hydroxymethil (CH_2_OH_(ad)_) as intermediate^56^. Ir has demonstrated a lower activity towards the MOR^57, 58^, and the reaction mechanism proposed on polycristalline Ir was the same as for Pt^57^. Nevertheless, the pH dependence of the rate of the MOR on Ir(111) and Pt(111) surfaces, together with the hereupon determined PME, E_PZTC_, OH adsorption potential, can elucidate the reaction mechanism and help tailoring the surface characteristic to achieve higher catalytic activity. The state-of-the-art anode materials for direct-methanol fuel cells (DMFC) are PtRu catalysts^59^. However, studies show a considerable decrease of the PtRu anode activity in a DMFC due to depletion of Ru^60, 61^. For this reason, PtIr catalysts are attracting considerable interest due to their higher stability and comparable activity with respect to PtRu^62–67^. Pt and Ir atoms can be combined to form solid solutions and enhance the activity towards the MOR due to their bifunctional effect. As demonstrated in this work, Ir strongly adsorb OH at lower potential than Pt, supplying oxygen species to the methanol adsorbed on the Pt atom, thus significantly promoting the MOR.

Perchloric and sulfuric acids are the commonly used supporting electrolytes for studies of the MOR. Nonetheless, the reaction can be affected by the anion of the supporting electrolyte used, as demonstrated for Pt(111), where a factor of ten higher current in HClO_4_ than in H_2_SO_4_ was reported^68^, due to the specific adsorption of sulfates anions on the surface. The comparison of electrochemical properties of the same crystallographic plane of Pt and Ir electrodes as a function of pH in HClO_4_ is potential tool to study the reaction in a simplified way. Furthermore, it allows the decoupling between geometric and electronic effect of the catalyst on the electrochemical properties and it permits the tailoring for the optimal Pt/Ir composition of the catalysts.

## Conclusions

Voltammetric profiles of Ir(111) in perchlorate containing solutions from pH = 1 to pH = 5 evidenced distinctive features, in the so-called H-upd region, which changed as the proton concentration increased. Voltammetric profiles of Ir(111) at pH = 1, pH = 2 and pH = 3 in sulfate containing electrolytes were also analyzed, revealing a shift of the sulfate adsorption peak of 30 mV per pH unit. The PME was assessed *via* LIPT measurements in perchlorate containing solutions from pH = 1 to pH = 5 and in sulfuric solutions from pH = 1 to pH = 3. The laser induced current transients in perchlorate containing solutions showed a PME shift of 30 mV per pH unit. Moreover, it locates within the H-upd region at lower electrode potential, i.e. E < 300 mV *vs* RHE, for all the pHs studied. This result strongly evidences that hydroxide-ions are likely adsorbed at ~0.25 V *vs* RHE in perchlorate containing solutions. This hypothesis has been confirmed by the E_PZTC_ value in perchlorate containing solutions with pHs from 1 to 4. The potential difference between PME and E_PZTC_ decreased as the proton concentration in the electrolytes decreased. Furthermore, the E_PZTC_ is higher than the PME, suggesting that at E_PZTC_ the surface excess of hydrogen is still higher than that of hydroxyl. Both LIPT and CO displacement experiments have shown the pivotal role of specific nature of the anion (either hydroxide or sulfate) in the redistribution of the charge across the interface.

The potential difference between the PME and *ϕ* ^*M*^ values of Ir(111) and Pt(111) evidences the large interfacial solvent contribution to the electrode surface potential. When the pH increases, even in absence of an electric field, the magnitude of the relative contribution of *g* ^*s*^(*dip*) and *δχ* ^*M*^ to the PME is described by a linear correlation having different slopes for Ir(111) and Pt(111), confirming the highest oxophilicity of Ir compared with Pt.

## Methods

An Ir(111) single crystal having diameter of 5 mm, surface roughness of 30 nm, and oriented better than 0.1° (Mateck) was employed in all experiments. Before each experiment, the Ir(111) electrode was annealed by means of inductive heating in a Ar (Ar 5.0, Air Liquide, Germany) + H_2_ (6.0, Air Liquide, Germany) atmosphere. The annealing and cooling were performed under a flow of Ar + H_2_ in the ratio 3:1, and the crystal was heated at a temperature higher than 1600 °C for 300 s. The energy was provided by an induction coil and its power supply (Hu 2000, Himmelwerk, Germany); the annealing temperature was checked by a pyrometer (IGA 140, Impac Infrared GmbH, Germany). The quartz tube was partially filled with ultra clean water in equilibrium with the H_2_/Ar gas mixture and the Ir(111) was transferred to the working cell protected with a drop of this water.

All chemicals employed in this work were of Suprapur^®^ quality. Electrolytes were prepared using HClO_4_ (Merck 70%, Suprapur, Germany), KClO_4_ (monohydrate, 99.99% Merck, Germany), and NaF (99.999%, Merck, Germany). The sulfate/sulfuric solutions were prepared using K_2_SO_4_ (99.999%, Merck, Germany), and H_2_SO_4_ (Suprapur 96%, Merck, Germany). Ultrapure water (18.2 MΩ cm) obtained from an Elgastat water purification system was used in all solutions. The pHs of the different solutions were measured using a PH-basic-20 pH-meter from Crison coupled with a pH-probed pH 50 12 HACH model.

To perform the LIPT measurements a four-electrode configuration was employed. Two Pt wires were used as a second working electrode and as a counter electrode, respectively. As a reference electrode, a Pd wire was employed, previously charged with H_2_ in a different compartment. The potentials recorded with the Pd/H_2_ reference electrode are shifted *ca* + 50 mV respect to the RHE electrode, but they were converted to either RHE or SHE scales and quoted in these scales in this paper. All the experiments were performed with the Ir(111) in the hanging meniscus configuration.

The procedure for recording the laser induced potential transients was described in detail elsewhere^69^. To summarize, before recording the transient, cyclic voltammetry was recorded using a μ-Autolab III potentiostat (Metrohm-Autolab, Utrecht, Netherlands) under the current integration mode, ensuring cleanness and stability of the surface. The second auxiliary electrode made of Pt was connected as an internal reference to measure the potential transients. At the beginning of each experiment, both working and second auxiliary electrode were polarized at the same potential. Approximately 200 μs before firing the laser both electrodes were disconnected from the potentiostat. The potential difference between both electrodes was measured under open circuit conditions. As the laser pulse only affects the single crystal working electrode, the measured potential difference is related to the response of the Ir(111)|aqueous solution interface. By applying short pulses of laser light the temperature of the interface rises suddenly and the change in the surface potential is measured at constant charge. The sign of this potential transient is mainly determined by the orientation of the water dipoles at the interphase.

Each experiment was repeated with a frequency of 10 Hz to ensure that the temperature relaxes to the initial value between the measurements. The potentiostat was reconnected between successive laser pulses to keep the potential at the desired value. In this way, 128 or 256 potential transients were recorded and averaged using a Tektronix Model TDS 3054B oscilloscope. Finally, after recording all potential transients, the cleanliness of the interphase was checked by recording a voltammetric profile.

The duration of the pulse was 5 ns, and the laser used was a 532 nm frequency (double harmonic) Nd-YAG (Brilliant B from Quantel). A conventional arrangement of mirrors directed the laser beam (6 mm) to the Ir(111)|aqueous solution interface. The cell was kept in a Faraday cage. The energy density of the laser beam was reduced to 3–5 mJ cm^−2^ by combining the effect of an attenuator from Newport Corporation (Model M-935-10) and the regulation of the Q-switch time. The laser energy was measured with a piroelectric sensor head (Model M-935-10).

The procedure to perform CO displacement measures is described in detail elsewhere^35, 70^. Before each CO measurement, the working electrode potential was held constant at the desired value and then a CO stream was allowed to flow. While the CO stream was dosed, a transient current was recorded until the whole Ir(111) surface was covered by a monolayer of CO. When the transient current fell to zero, CO flow was stopped and Ar was bubbled into the solution for 10 min to remove the excess CO in the cell. Then the CO monolayer was stripped electrochemically. For both the cyclic voltammograms and the amperometric measurements, a waveform generator (EG&G PARC 175) together with a potentiostat (eDAQ EA161) and a digital recorder (eDAQ ED401) were employed.

## Electronic supplementary material

Supplementay
