# Supplementary material for: On the pH Dependence of the Potential of Maximum Entropy of Ir(111) Electrodes
Source: Sci Rep. 2017 Apr 28;7:1246. doi: 10.1038/s41598-017-01295-1 (PMC5430915; doi:10.1038/s41598-017-01295-1)
Supplement: Supplementary file 1 — Supplementay [file 41598_2017_1295_MOESM1_ESM.pdf]

Supplementary information

On the pH Dependence of the Potential of Maximum Entropy of Ir(111) Electrodes

Alberto Ganassin [1], *Paula Sebastián* [2], *Víctor Climent* [2], *Wolfgang Schuhmann* [1],

Aliaksandr S. Bandarenka [3,*], *Juan Feliu* [2,*]

1 *Analytical Chemistry - Center for Electrochemical Sciences (CES); Ruhr-Universität Bochum, Universitätsstr. 150, 44780 Bochum, Germany*

2 *Instituto de Electroquímica, Universidad de Alicante, Apartado 99, E-03080 Alicante, España-Spain*

3 *Energy Conversion and Storage – ECS, Physik-Department, Technische Universität München, James-Franck-Straße 1, 85748 Garching, Germany*

*Corresponding authors, e-mails:

bandarenka@ph.tum.de, tel. +49 (0) 89 289 12531 (A.S. Bandarenka)

juan.feliu@ua.es , tel. +34 965 909 301 (J. Feliu)

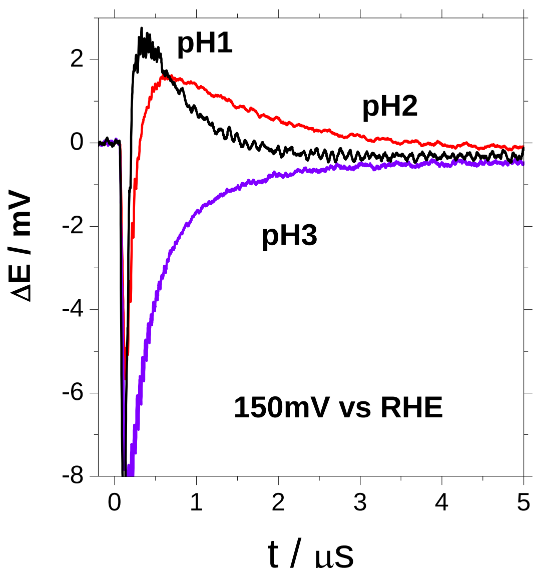

Figure S1: Laser induced potential transients of Pt(111) recorded at 150 mV vs RHE in Ar-saturated solution of:.0.1 M $HClO_4$ (black line, pH=1), 0.01 M $HClO_4$ + 0.099 M $KClO_4$ (red line, pH=2) and 1 mM $HClO_4$ + 0.1 M $KClO_4$ (purple line, pH=3).

Determination of the thermal coefficients $(dE/dT)_q$ from the laser induced E-t transients:

$$\Delta E=\frac{1}{2}\left(\frac{\partial E^M}{\partial T}\right)_q T_0\sqrt{\frac{t}{t_0}} \qquad (1)$$

Where E^M is the electrode potential, T_0 is the temperature, t is the time, and q is the electrode charge. From the Electrocapillary equation it can be shown that $(dE^M/dT)_q$ is equivalent to $(d\Delta S/dq)_T$ in the absence of specific adsorption, where ΔS is the entropy of formation of the double layer [1,2]. The thermal coefficient is extracted from the laser induced potential transient data by the following graphic procedure. If the reorganization of the double layer is fast enough in the time scale employed, and the change in the temperature is small, then the potential change

ΔE should vary linearly with the square root of time, according to equation (1). Therefore, the thermal coefficient can be extracted from the slope of these plots:

$$\frac{\partial E^M}{\partial T} = \frac{2*\text{slope}}{\Delta T_0 \sqrt{t_0}} \left(\text{mV K}^{-1}\right) \qquad (2)$$

The maximum temperature change ΔT_0 can be calculated from the optical properties of the surface and the thermal properties of the adjacent phases. For Ir(111) the maximum temperature change reached is 25°C for a laser beam energy of 1 mJ.

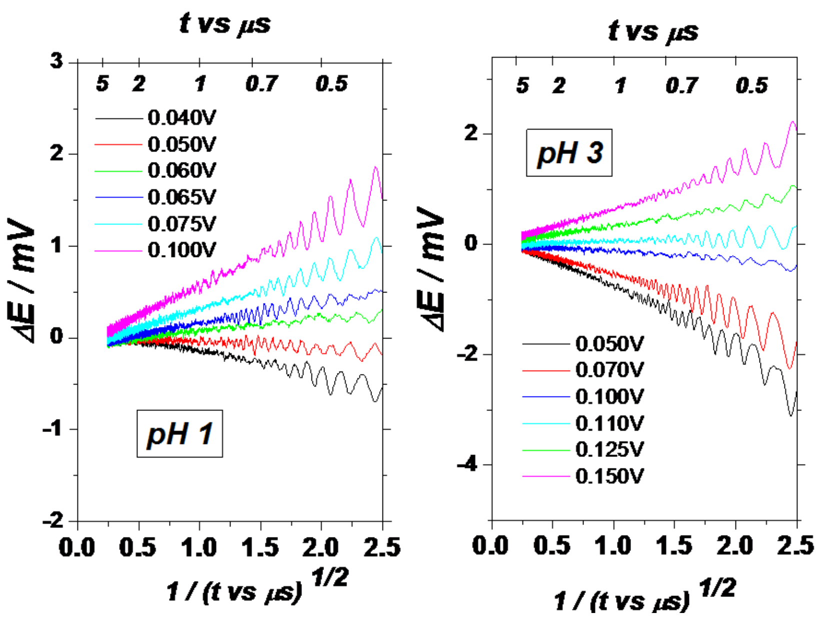

Figure S2: Dependencies of the electrode potential on the inverse of the square root of the time of the transients recorded at pH=1 and pH=3 in perchloric solutions whose composition is as described in the manuscript (Figure 1).

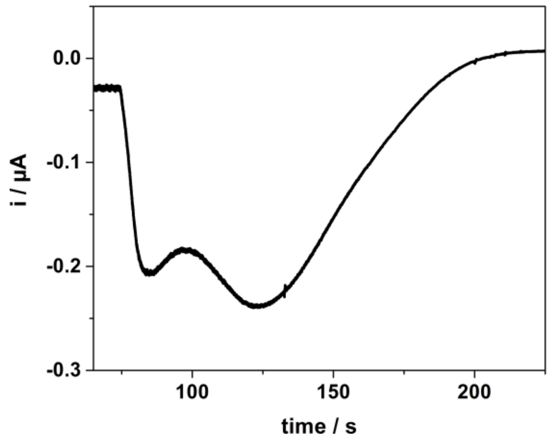

Figure S3: The results of the CO displacement experiment on Ir (111) in 0.1 M $HClO_4$ at 0.3 V vs. RHE. Negatively charged species are displaced from the surface at potentials more positive than the anodic peak at ~0.25V vs RHE observed in cyclic voltammetry in Figure 2 of the manuscript.

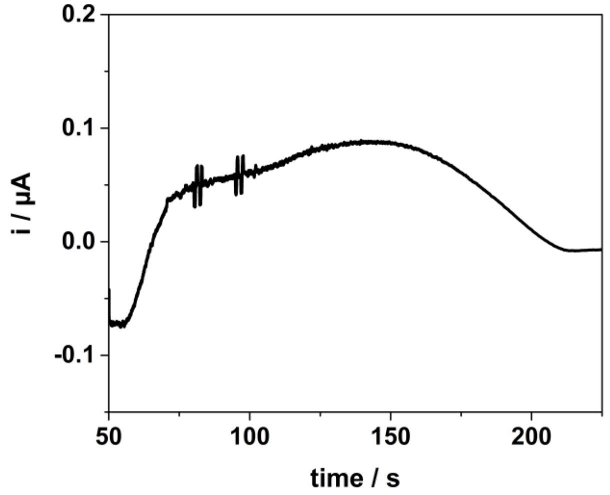

Figure S4: The results of the CO displacement experiment using Ir (111) electrodes in contact with 0.1 M $HClO_4$ at 0.15 V vs. RHE. Positively charged species are displaced from the surface at potentials more negative than the anodic peak at ~0.25 V vs RHE as observed in the voltammograms shown in Figure 2 of the manuscript.

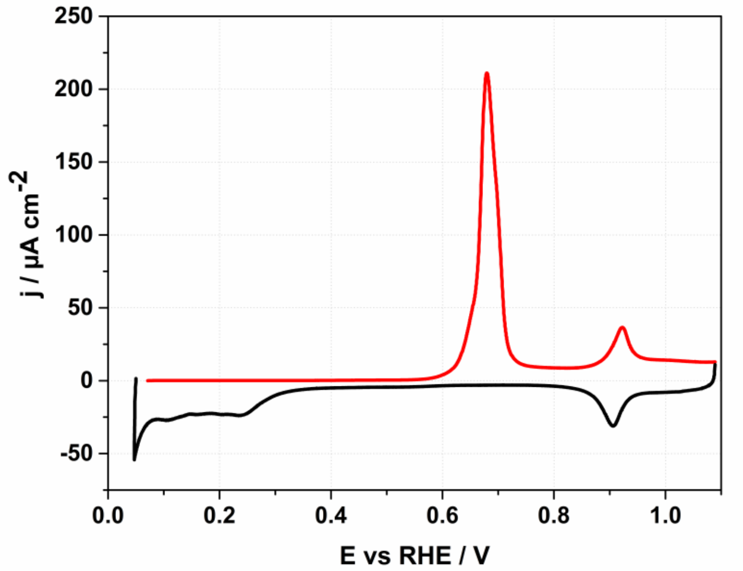

Figure S5: Typical CO stripping voltammogram obtained for Ir(111) in 10 mM $HClO_4$ + 0.09 M $KClO_4$ (pH=2) electrolytes at 0.3 V vs RHE. Red line represents the CO stripping and the black line shows the cathodic scan after the stripping. Scan rate 20 mV/s.

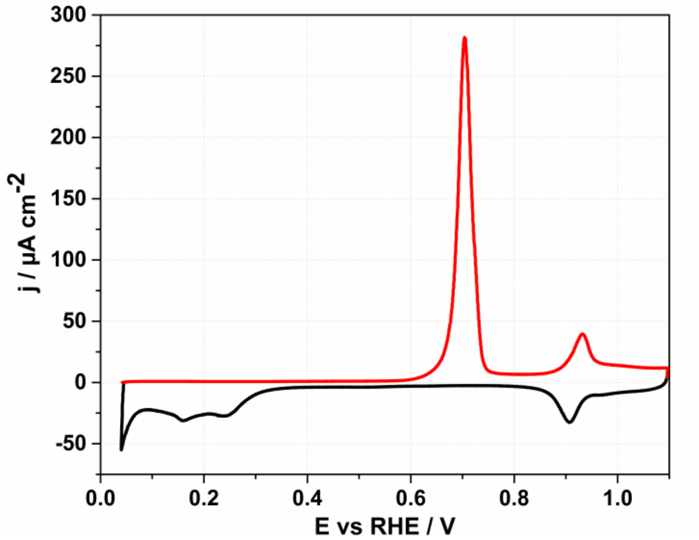

Figure S6: Typical CO stripping voltammogram obtained in 1 mM $HClO_4$ + 0.1 M $KClO_4$ (pH=3) for Ir(111) electrodes after the CO displacement at 0.3 V vs RHE. Red line represents the CO stripping and the black line shows the cathodic scan after the stripping. Scan rate 20 mV/s.

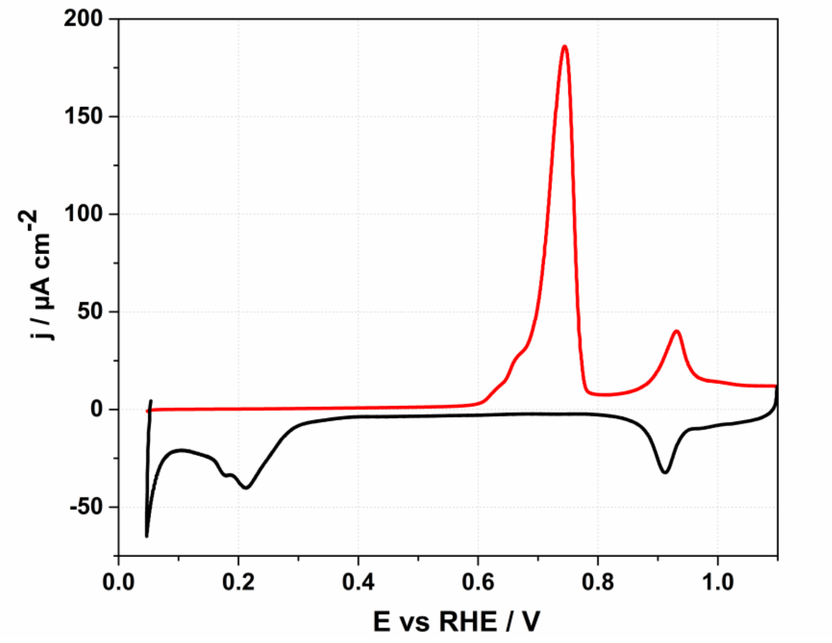

Figure S7: CO stripping in 0.08 M $KClO_4$ + 0.02 M NaF/HF (pH=4) after the CO displacement at 0.3 V vs RHE. Red line represents the CO stripping and the black line the cathodic scan after stripping. Scan rate 20 mV/s.

References

[1] N. Garcia-Araez, V. Climent, J. M. Feliu, in *Modern aspects of electrochemistry*, Vol. 51 (Ed: C. G. Vayenas), Springer, New York **2011**, pp. 1–103.

[2] V.A. Benderskii, G.I. Velichko, *J. Electroanal. Chem. Interfacial Electrochem.* **1982**, *140*, 1.
